# Interfacial “Anchoring Effect” Enables Efficient Large‐Area Sky‐Blue Perovskite Light‐Emitting Diodes

**DOI:** 10.1002/advs.202102213

**Published:** 2021-08-28

**Authors:** Yang Shen, Jing‐Kun Wang, Yan‐Qing Li, Kong‐Chao Shen, Zhen‐Huang Su, Li Chen, Ming‐Lei Guo, Xiao‐Yi Cai, Feng‐Ming Xie, Xiao‐Yan Qian, Xingyu Gao, Ivan S. Zhidkov, Jian‐Xin Tang

**Affiliations:** ^1^ Jiangsu Key Laboratory for Carbon‐Based Functional Materials & Devices Institute of Functional Nano & Soft Materials (FUNSOM) Soochow University Suzhou 215123 China; ^2^ School of Physics and Electronic Science Ministry of Education Nanophotonics & Advanced Instrument Engineering Research Center East China Normal University Shanghai 200062 China; ^3^ Key Laboratory of Interfacial Physics and Technology Shanghai Synchrotron Radiation Facility Zhangjiang Laboratory Shanghai Institute of Applied Physics Chinese Academy of Sciences Shanghai 201204 China; ^4^ Institute of Physics and Technology Ural Federal University Mira 19 str. Yekaterinburg 620002 Russia; ^5^ Macao Institute of Materials Science and Engineering (MIMSE) Macau University of Science and Technology Taipa Macau SAR 999078 China

**Keywords:** anchoring effect, crystallization manipulation, interface engineering, perovskite light‐emitting diodes

## Abstract

While tremendous progress has recently been made in perovskite light‐emitting diodes (PeLEDs), large‐area blue devices feature inferior performance due to uneven morphologies and vast defects in the solution‐processed perovskite films. To alleviate these issues, a facile and reliable interface engineering scheme is reported for manipulating the crystallization of perovskite films enabled by a multifunctional molecule 2‐amino‐1,3‐propanediol (APDO)‐triggered “anchoring effect” at the grain‐growth interface. Sky‐blue perovskite films with large‐area uniformity and low trap states are obtained, showing the distinctly improved radiative recombination and hole‐transport capability. Based on the APDO‐induced interface engineering, synergistical boost in device performance is achieved for large‐area sky‐blue PeLED (measuring at 100 mm^2^) with a peak external quantum efficiency (EQE) of 9.2% and a highly prolonged operational lifetime. A decent EQE up to 6.1% is demonstrated for the largest sky‐blue device emitting at 400 mm^2^.

## Graphical abstract

A facile and effective grain‐growth manipulation scheme enabled by interface “anchoring effect” is proposed for high‐quality sky‐blue perovskite emitter. Remarkable boost in device performance is achieved for large‐scale sky‐blue perovskite LEDs with peak external quantum efficiencies of 9.2% and 6.1% measured at scales of 100 and 400 mm^2^, respectively.

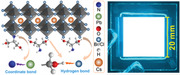

## 1 Introduction

Perovskite light‐emitting diodes (PeLEDs) are emerging as a promising candidate for the applications of high‐quality full‐color displays due to their tunable emissions, high color purity, and simple solution processability.^[^ ^1^, ^2^, ^3^, ^4^, ^5^ ^]^ In recent years, great breakthroughs have been witnessed in external quantum efficiencies (EQEs), which exceed 20% with emission colors ranging from green to near‐infrared.^[^ ^6^, ^7^, ^8^ ^]^ However, as one of the primary‐color devices, the performance of blue PeLEDs still lags far behind from the other counterparts in terms of device efficiency and operational stability.^[^ ^9^, ^10^, ^11^ ^]^ Meanwhile, all the reported device areas of state‐of‐the‐art blue PeLEDs remain small (at several mm^2^) due to uneven morphologies and vast defects in the solution‐processed mixed halide (e.g., Br and Cl) perovskite films,^[^ ^12^, ^13^ ^]^ and large‐area blue PeLEDs have not yet been reported to date.

It has been widely recognized that the increase in film sizes gives rise to the significant performance loss for perovskite optoelectronic devices. Prominent boundaries or crevices between perovskite domains are inevitable in large‐area solution‐processed perovskite films due to nonuniform coating, limited precursor solubility and thermal convection during the solvent evaporation.^[^ ^14^ ^]^ The inextricable efficiency roll‐off occurs in the enlarged perovskite devices. This issue has severely hindered the practical use in large‐area displays since the stochastic occurrence of pinholes or defective sites inevitably gives rise to severe trap‐mediated nonradiative recombination loss and the broad variation in luminescent performance across multiple device pixels.^[^ ^15^, ^16^, ^17^ ^]^ Furthermore, the degradation of blue PeLEDs will be accelerated because the defects can act as the hoping sites for ion migration under external bias.^[^ ^18^ ^]^

To produce uniform perovskite films with suppressed trap‐states, various strategies have been proposed, including stoichiometric‐composition engineering, dimensional manipulation, A‐site cation doping, Lewis‐base passivation and so on.^[^ ^11^, ^12^, ^13^, ^19^, ^20^, ^21^ ^]^ Besides, the interface between perovskite films and the underlying hole‐transport layer (HTL) has a strong influence on the perovskite crystallization behaviors and electrical properties.^[^ ^22^, ^23^, ^24^ ^]^ Motivated by this issue, we propose an interface engineering method by using a multi‐functional organic molecule 2‐amino‐1,3‐propanediol (APDO) to manipulate the grain growth of sky‐blue perovskite films and to alleviate the hole‐injection efficiency and charge balance. The APDO‐modified HTL‐perovskite interface promotes the 2D and small‐size perovskite grains with large‐area uniformity and low trap‐state density, resulting from the functional group‐triggered “anchoring effect.” Eventually, large‐area sky‐blue PeLED (measuring at 100 mm^2^) achieves a peak EQE of 9.2% and a maximum luminance of 1775 cd m^−2^, respectively. Furthermore, larger‐area devices (at 400 mm^2^) operate with a decent EQE up to 6.1% using the same strategy.

## 2 Results and Discussion

### 2.1 Interface‐Triggered Perovskite Crystallization

**Figure 1:**
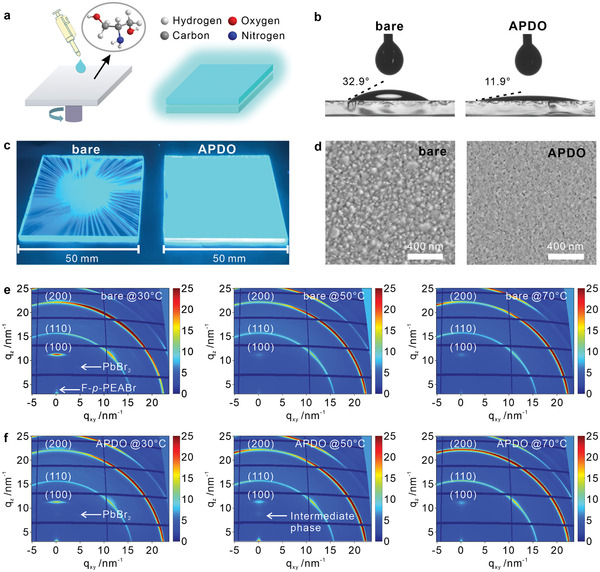
Growth of perovskite films on bare and APDO‐modified PEDOT:PSS. a) Schematic of the interfacial modification with multi‐functional molecule APDO. b) Contact angles of perovskite precursor droplets on different substrates. c) Photographs of large‐area perovskite films (50 mm × 50 mm) under UV lamp. d) Top‐view SEM images of perovskite films on different substrates. GIWAXS patterns of perovskite films on e) bare and f) APDO‐modified PEDOT:PSS annealed for 3 min at 30, 50, and 70 °C, respectively.

The grain‐growth interface has profound influences on the perovskite crystallization and interfacial carrier transfer process. Inspired by previous reports that hydroxyl (—OH) and amine (—NH_2_) groups can effectively interact with perovskites and passivate defects,^[^ ^25^, ^26^, ^27^, ^28^, ^29^ ^]^ we anticipate that proper additives with such organic groups may induce exciting manipulation in perovskite film formation and hence influencing its photophysical properties. Herein, a small and water‐soluble organic molecule of APDO with both —OH and —NH_2_ groups was employed to modify the HTL of poly(3,4‐ethylene dioxythiophene):polystyrene sulfonic acid (PEDOT:PSS) (see the details in the Experimental Section). **Figure** **1**a illustrates the schematic progress of preparing the mixed halide CsPb(Br/Cl)_3_ sky‐blue perovskite films with the APDO‐based interface modification. X‐ray photoelectron spectroscopy (XPS) characterization verifies the existence of APDO in the modified PEDOT:PSS HTL (Figure S1, Supporting Information). Moreover, to examine the APDO distribution in PEDOT:PS layer, the angle‐dependent XPS measurements were conducted to calculate the ratios (N/S) of the peak area from N 1s to that from S 2p at 70° and 90°, respectively. The peak areas integrated from the XPS spectra can be used for comparing the contents of various elements. Consequently, the N/S ratio at 70° is higher than that at 90°, indicating that APDO is preferentially distributed on the top of the PEDOT:PSS HTL since N *1s* signal comes from the APDO molecules (Figure S2 and Table S1, Supporting Information). A remarkable decrease in contact angle from 32.9° to 11.9° is observed for perovskite precursor droplets on bare and APDO‐modified PEDOT:PSS HTLs (Figure 1b), revealing the improved wetting properties of the APDO‐modified substrate for the perovskite growth. The resulting sky‐blue perovskite film deposited on APDO‐modified PEDOT:PSS HTL displays an improved film coverage for a large‐area substrate with a size of 50 mm × 50 mm (Figure 1c and Figure S3, Supporting Information). Figure 1d displays the top‐view scanning electron microscopy (SEM) images of sky‐blue perovskite films, microscopically depicting the more uniform morphology of the APDO‐modified perovskite film with smaller grain sizes than that on the bare substrate. As determined by atomic force microscopy (AFM) characterization (Figure S4, Supporting Information), the root mean square (rms) roughness of sky‐blue perovskite films is significantly decreased from 3.44 nm to 1.47 nm through the APDO‐induced interface modification.

To gain deep insight into the crystallization kinetics of perovskite films, grazing‐incidence wide‐angle X‐ray scattering (GIWAXS) characterization was conducted to investigate the morphological evolution. Figure 1e,h compares the 2D GIWAXS patterns of sky‐blue perovskite films on bare and APDO‐modified PEDOT:PSS HTLs, in which three main lattice planes (i.e., (100), (110), and (200) faces) can be identified for CsPb(Br/Cl)_3_ perovskites. It is noted that the lead bromide (PbBr_2_) scattering ring is obvious for perovskites annealed at 30 °C on both bare and APDO‐modified substrates, which indicates the typically incomplete crystallization process at a low temperature. Apart from these patterns, a punctate scattering peak at a small *q*_z_ value appears in the perovskite film formed on bare PEDOT:PSS, which results from the contribution of 4‐Fluorophenylethylammonium bromide (*p*‐F‐PEABr). However, the *p*‐F‐PEABr scattering ring vanishes through the APDO‐induced interface engineering, which is expected to be favorable for the control of perovskite phase separation and grain size distribution.^[^ ^30^ ^]^ When the annealing temperature is elevated to 50 °C, the PbBr_2_ scattering pattern disappear in both cases. Meanwhile, it is evident that a non‐negligible scattering signal of intermediate 2D perovskite phase appears in the APDO‐modified perovskite film, implying that APDO plays an important role in promoting the formation of 2D perovskite phases. At an annealing temperature of 70°C, only the scattering rings from (100), (110), and (200) planes can be found, indicating the complete crystallization process for perovskite films under two conditions. The comparison of 2D GIWAXS patterns reveals the better crystallinity of perovskite grains in the APDO‐modified perovskite film. The bolder scattering rings of the lattice planes in APDO‐modified perovskite film are in great accordance with the reduced perovskite grain sizes as displayed in the SEM images (Figure 1d).

The photophysical properties of the perovskite films formed on different substrates are compared (Figure S5, Supporting Information). The steady‐state photoluminescence (PL) spectra reveal a small blue‐shift from 490 nm of the perovskite film on bare PEDOT:PSS to 488 nm of the sample on APDO‐modified substrate, resulting from the enhanced formation of 2D perovskites with APDO‐induced interface engineering as discussed above. It is observed that a distinct improvement of PL intensities and transient PL lifetime is obtained for the perovskite film formed on the APDO‐modified PEDOT:PSS HTL. It is well known that the improvement of transient PL lifetimes is ascribed to the reduction of trap‐mediated nonradiative recombinations.^[^ ^18^, ^24^ ^]^ In addition, the photoluminescence quantum efficiencies (PLQYs) of perovskite films with various APDO‐doping concentrations are collected in Figure S6 (Supporting Information) for comparison. The PLQYs of APDO‐modified samples show much higher values than that of the control one, providing a direct evidence for the passivation effect of the interface engineering.^[^ ^31^, ^32^ ^]^

### 2.2 Device Performance of Large‐Area PeLEDs

**Figure 2:**
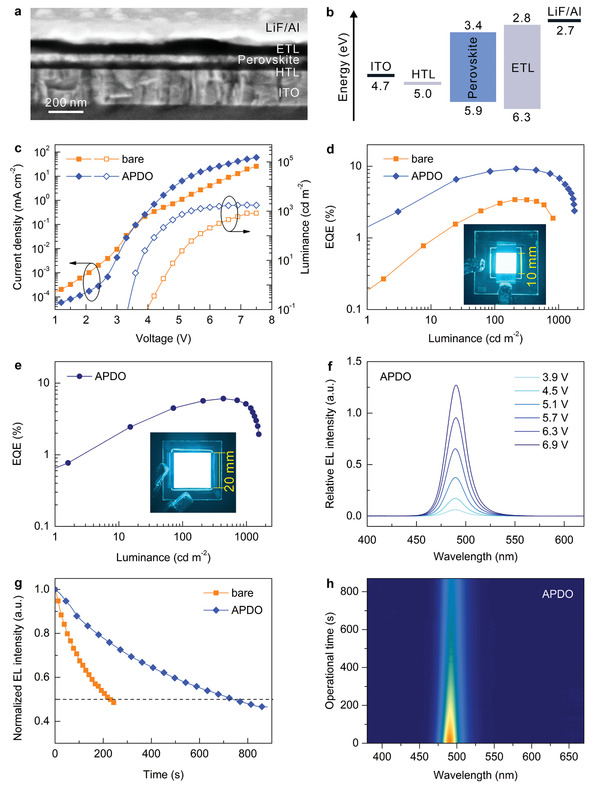
Device structure and performance of large‐area sky‐blue PeLEDs. a) Cross‐sectional SEM image of the sky‐blue PeLED. b) Schematic energy‐level diagram of the device. c) Current density–voltage–luminance (*J*–*V*–*L*) characteristics. d) EQE versus luminance. The insets are the photographs of the operating devices (100 mm^2^). e) EQE versus luminance for large‐area APDO‐modified device (400 mm^2^). Inset is the photograph of the corresponding device. f) EL spectra of the APDO‐modified PeLED at various bias voltages. g) Operational lifetime curves at an initial luminance of 100 cd m^−2^. h) The evolution of EL spectra under continuous operation for the APDO‐modified PeLED.

Driven by the remarkable improvement of morphological and photophysical characteristics of APDO‐modified perovskite films, large‐area sky‐blue PeLEDs were fabricated with an architecture of indium tin oxide (ITO)/APDO‐modified PEDOT:PSS/perovskite/1,3,5‐Tris(1‐phenyl‐2‐benzimidazolyl)benzene (TPBi)/lithium fluoride (LiF)/aluminum (Al). Here, TPBi was used as an electron‐transport layer (ETL), and LiF/Al functioned as a bilayer cathode. The detailed device performance of PeLEDs is summarized in **Table** **1**. **Figure** **2**a displays the cross‐sectional SEM image of the device. The schematic energy‐level diagram of the sky‐blue PeLED is shown in Figure 2b, in which the highest occupied molecular orbital (HOMO) and lowest unoccupied molecular orbital (LUMO) of the perovskite film as well as TPBi were determined from ultraviolet photoelectron spectroscopy (UPS) and absorption measurements (Figures S7 and S8, Supporting Information). It is found that the APDO‐induced interface engineering can give rise to the negligible change in the valance and conduction bands of perovskite films. Figure 2c plots the current density–voltage–luminance (*J*–*V*–*L*) curves of the APDO‐modified PeLED with an emitting area of 100 mm^2^ (also see Video S1, Supporting Information). Compared to the control device with a bare HTL, the APDO‐modified PeLED exhibits a remarkably reduced turn‐on voltage (*V*_on_, defined as the driving voltage at a luminance of 1 cd m^−2^) and the improved electrical properties. For example, the leakage current of the APDO‐modified device is suppressed under the bias lower than Von, while its current density is enhanced under the high bias condition. The suppressed leakage current is mainly on account of the reduced nonradiative defects at the interface between the HTL and perovskite and the improved perovskite film coverage enabled by the compact and uniform small grains. With the APDO‐induced interface engineering, the peak luminance and EQE of large‐area sky‐blue PeLED reach 1775 cd m^−2^ and 9.2%, which are significantly larger than that of the control device with a peak luminance of 819 cd m^−2^ and EQE of 3.4% (Figure 2d). The previously reported representative blue PeLEDs have been summarized in Table S4 (Supporting Information), indicating that the champion large‐scale device in this work can be comparable to those state‐of‐the‐art ones that features small emitting area in device performance. Even when the emitting area is extended to 400 mm^2^ (also see Video S2, Supporting Information), sky‐blue PeLED with decent performance can be achieved using the same strategy, showing a record high EQE of 6.1% (Figure 2e). The angular distribution of the electroluminescence (EL) intensity shows that the emission patterns of both the control and APDO‐modified devices are almost identical to an ideal Lambertian profile (Figure S9, Supporting Information), and the perceived EL spectra are independent of viewing angle. Transient EL decay measurements were also performed to compare the response characteristics of the bare and APDO‐modified PeLEDs under an electrical pulse. The faster EL response to the electrical pulse can be found for the APDO‐modified device than that of the bare one (Figure S10, Supporting Information), revealing the reduced trap states and improved hole transport in the APDO‐modified PeLED.^[^ ^23^, ^33^ ^]^

**Table 1:** Device performance of large‐area sky‐blue CsPbBr_3−_ *_x_* Cl*_x_* PeLEDs with and without APDO‐induced interface engineering

Device types	Area [mm^2^]	*V*_ON_ [V]	EL peak [nm]	Max *L* [cd m^−2^]	EQE [%]	*T*_50_ ^a)^ [s]
Bare	100	4.3	492	826	3.4	224
APDO‐modified	100	3.5	490	1775	9.2	740
APDO‐modified	400	3.5	490	1564	6.1	578

^a)^ T_50_ data were measured at an initial luminance of 100 cd m^−2^.

Furthermore, there is no perceivable change in the EL spectra of the APDO‐modified devices when increasing the bias voltage (Figure 2f), showing the bias‐independent luminescence stability. The APDO‐modified PeLED features a distinctly enhanced operational half‐lifetime (*T*_50_, at which the luminance declines to 50% of its initial value) as compared to that of the control device (Figure 2g). As summarized in Table 1, the *T*_50_ of the APDO‐modified device with an area of 100 mm^2^ is ≈740 s, which is almost 4 times of the control device (*T*_50_ ≈ 224 s) at an initial luminance of 100 cd m^−2^. Considering the less defect‐tolerance feature of Cl‐contained perovskites and severe carrier transfer imbalance of blue PeLEDs, the enhanced operational lifetime of large‐area PeLEDs is ascribed to the suppressed nonradiative recombination and improved electrical properties caused by the APDO‐induced interface engineering. It is also noted that the EL spectra of the APDO‐modified device remain almost unchanged during the continuous operation measurement as collected in Figure 2h. The high stability of EL spectra in APDO‐modified device can be attributed to the reduced defects in perovskite films, which can behave as the main channel for halide ion migration and phase segregation, along with facilitating device degradation under external stimulations.^[^ ^18^ ^]^

### 2.3 “Anchoring Effect” of APDO‐Induced Interface Engineering

**Figure 3:**
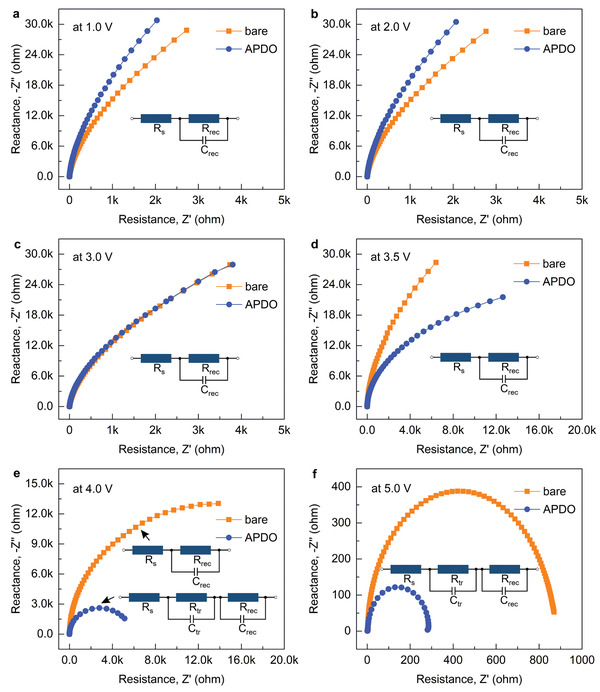
Carrier recombination dynamics in large‐area PeLEDs. Impedance spectra under an AC signal with an amplitude of 300 mV for the devices operated with a DC bias of a) 1.0 V, b) 2.0 V, c) 3.0 V, d) 3.5 V, e) 4.0 V, and f) 5.0 V, respectively. Insets are the equivalent circuit models.

To identify the origin of the performance improvement enabled by the APDO‐induced interface engineering, impedance spectroscopy (IS) measurements were conducted to examine the charge transport and recombination behaviors. **Figure** **3** plots the semicircle curves of the Nyquist plot of large‐area PeLEDs at various bias voltages. The equivalent circuit models consisting of capacitor, parallel and series resistors are applied for analyzing the Nyquist plot, where the composite reactance (*R*_rec_) and resistance (*R*_s_) are associated with the recombination rate and contact resistance in PeLEDs, respectively.^[^ ^34^ ^]^ It is noted that the IS curves maintain a similar tendency under the nonemission stage at low forward voltages (<3.0 V) (Figure 3a,b), which were analyzed using a simple equivalent circuit model comprising a parallel capacitor–resistor (CR) unit in series with a resistor (*R*_s_). The *R*_s_ values were estimated to be around 3.2 Ω cm^2^ and 1.9 Ω cm^2^ for bare and APDO‐modified PeLEDs, respectively. The fitted *R*_rec_ values are summarized in Table S3 (Supporting Information), showing that the APDO‐modified PeLED exhibits higher *R*_rec_ values than those of the bare device. As the APDO modification has negligible influence on the work‐function and hole‐transport capability of the PEDOT:PSS (Figure S11, Supporting Information), the contribution of the HTLs to the IS curves can be ruled out. According to the previous reports,^[^ ^35^, ^36^ ^]^ the high *R*_rec_ and low *R*_s_ at low bias in the APDO‐modified device can be ascribed to the improved interface contact between the HTL and perovskite films and thus the suppressed nonradiative recombination triggered by the effective APDO‐induced crystallization manipulation. The abnormal variation is observed for the IS curves when the bias voltage is further increased (Figure 3c–f). When the bias voltage is increased to 3.0 V, the nearly identical curves are collected for both bare and APDO‐modified PeLEDs (Figure 3c), representing a turning point of the competition between trap‐mediated nonradiative recombination and radiative recombination. It is worth noting that the distinct shrink of *R*_rec_ values occurs beyond Von in both bare and APDO‐modified devices (Figure 3d–f), revealing that the built‐in potential barriers at the HTL/perovskite interfaces have been overcome and the electron‐hole recombination is accelerated to higher level under the more intense electric filed. It is also noted that an extra parallel RC unit is added in the equivalent circuit, which represents the carrier transfer in transport layers under light‐emission stage in open circuit.^[^ ^37^ ^]^ Apparently, the *R*_rec_ of the APDO‐modified PeLED decreases more remarkably as compared with that of the bare device as the bias voltage goes beyond 3.5 V (Figure 3d–f), implying the higher recombination rate enabled by the APDO‐induced interface engineering. This transition undoubtedly provides a strong evidence for the enhanced hole transport from the APDO‐modified HTL to perovskite emitter since the same ETL is used.^[^ ^38^ ^]^ Accordingly, the charge transport balance within the radiative recombination zone is improved, which is in good agreement with the distinctly elevated device performance as discussed above.

**Figure 4:**
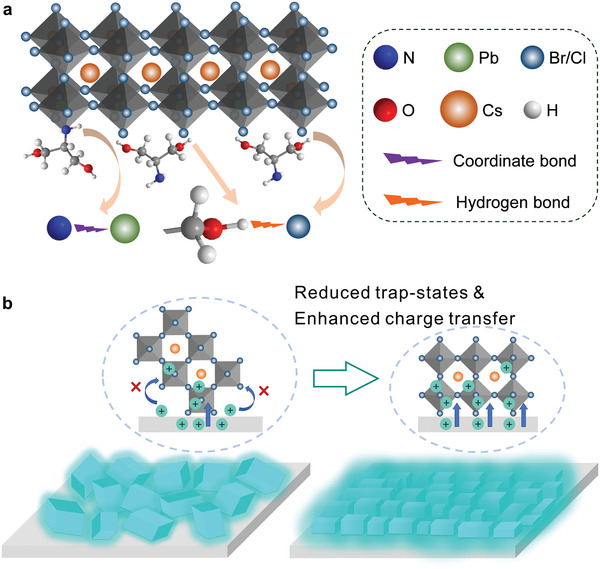
Schematic of APDO‐induced “anchoring effect” on the perovskite film. a) Perovskite grain growth and trap‐state passivation with APDO. b) Hole‐transport characteristics from bare and APDO‐modified PEDOT:PSS HTLs to perovskite films.

In accordance with the above accumulated evidence, it is reasonably inferred that the APDO‐induced interface engineering plays a vital role in the grain growth of large‐area perovskite emitters and the performance improvement of sky‐blue PeLEDs. **Figure** **4** illustrates the mechanisms of the morphological control over large‐area perovskite films with the APDO‐induced interfacial modification. The effective “anchoring effect” may be triggered by the APDO during the perovskite crystallization process. As demonstrated by the XPS characterization (Figure S2 and Table S1, Supporting Information), the APDO is preferentially distributed on the surface of the modified PEDOT:PSS HTL. During the continuous crystallization process of CsPb(Br/Cl)_3_ perovskite from the precursor solution on the APDO‐modified PEDOT:PSS HTL, the ‐OH group in APDO tends to form the hydrogen bonding with the halide anions (Br^−^/Cl^−^) at the perovskite grain‐growth interface, which has been verified by the measurements of ^1^H nuclear magnetic resonance and Fourier transform infrared spectroscopy (Figure S12 and S13, Supporting Information).^[^ ^25^, ^29^, ^39^, ^40^ ^]^ As depicted in Figure 4a, such an interaction between the modified PEDOT:PSS and perovskite holds great potential in providing masses of nucleation seeds tightly linked onto the grain‐growth substrate for preferential crystallization of PbX_2_ octahedrons (X = Br/Cl). The resulting perovskite film exhibits much more uniform and pinhole‐free morphology with smaller grain sizes (see in Figure 1d). In addition, it has been widely recognized that —NH_2_ groups can suppress the uncoordinated Pb‐induced fluorescence quenching centers through coordinate bond interaction.^[^ ^26^, ^27^, ^28^ ^]^ The XPS characterization reveals the coordination coupling between ‐NH_2_ groups and uncoordinated Pb atoms at the bottom of the perovskite emitter (Figure S14, Supporting Information). Meanwhile, the interaction between the APDO molecules and perovskite may behave as the pulling force between precursor solution and APDO‐modified PEDOT:PSS layer, contributing to more hydrophilic surface with the increased nucleation seeds. Figure S15 (Supporting Information) displays the schematic process of perovskite film formation on bare and APDO‐modified substrates, providing a direct demonstration for the film morphology evolution in a relatively more macroscopic view. To be specific, smaller and denser perovskite crystal grains can be obtained to form a compact film as the wettability of grain‐growth interface is improved. The “anchoring effect” triggered by —OH and —NH_2_ groups of APDO molecules collectively contributes to the improved perovskite morphology and interfacial defect passivation, significantly boosting the radiative recombination of large‐area perovskite emitter. Furthermore, Figure 4b vividly describes the hole‐transport process in the conditions with and without APDO. Compared with the randomly distributed perovskite grains with larger sizes on bare PEDOT:PSS HTL, the APDO‐modified perovskite film is assembled by well‐packed grains, achieving more efficient hole injection and transport. It is well known that the less efficient and imbalanced injection of holes in blue PeLEDs is a critical factor limiting the device performance,^[^ ^6^, ^22^, ^41^, ^42^ ^]^ and a large quantity of excessive electrons are lost at the interface near the HTLs through nonradiative recombination.^[^ ^43^, ^44^ ^]^ The promoted injection and transport of holes at the APDO‐modified perovskite/HTL interfaces can definitely ameliorate the utilization ratio of injected carriers and enhance the device efficiency as shown in Figures 2 and 3. Consequently, the “anchoring effect” caused by the APDO‐induced interface engineering is capable of simultaneously promoting the morphological control, reducing the trap‐mediated nonradiative recombination in perovskite emitter, and enhancing the hole transport inside the device.

## 3 Conclusion

In summary, a facile and effective interface engineering strategy has been demonstrated to boost the luminescent performance of large‐area sky‐blue perovskite films and PeLEDs. The “anchoring effect” triggered by a multi‐functional APDO molecule enables the formation of large‐area perovskite emitters with improved morphological uniformity and efficient radiative recombination. The trap‐mediated nonradiative recombination is suppressed, and the hole‐transport capability is enhanced in the APDO‐modified perovskite film. Through this rational interface engineering, large‐area sky‐blue PeLEDs measuring at 100 mm^2^ achieve a high EQE of 9.2% and a significantly prolonged operational lifetime. The peak EQE maintains a decent value of 6.1% even when the emitting area is extended to 400 mm^2^. Our strategy intending for efficient and stable large‐area sky‐blue PeLEDs may provide a promising guide toward high‐quality displays and other kinds of optoelectronic applications.

## 4 Experimental Section

### Materials

Cesium bromide (CsBr, 99.0%), cesium iodide (CsI), lead bromide (PbBr_2_, 99.0%), lead iodide (PbI_2_), and lead chloride (PbCl_2_, 99.0%) were purchased from TCI. Potassium bromide (KBr, 99.5%) and APDO were purchased from Aladdin Industrial Corp. Dimethyl sulfoxide (DMSO) (99.7%) and lithium fluoride (LiF, 99.99%) were purchased from MACKLIN. 1,3,5‐Tris(1‐phenyl‐2‐benzimidazolyl)benzene (TPBi, 99.0%) was purchased from Nichem. 4‐Fluorophenylethylammonium bromide (p‐F‐PEABr, 99.5%) was purchased from Xi'an Polymer Light Technology Corp. PEDOT:PSS (Clevios Al 4083) was purchased from Heraeus. All these chemicals were directly used without further purification.

### Preparation of Perovskite Films

The sky‐blue perovskite precursor solution was prepared in a nitrogen‐filled glove box by dissolving CsBr, PbBr_2_, PbCl_2_, p‐F‐PEABr in DMSO solvent with a molar ratio of 14:5:5:4, in which the concentration of CsBr was fixed at 0.19 m. The KBr with a concentration of 4 mg mL^−1^ was also incorporated as a passivation additive. The precursor solution was stirred at 40 °C for 4 h. Then, the perovskite films were prepared in the glove box by spin‐coating the precursor solution on the PEDOT:PSS‐coated substrate at 3000 rpm for 60 s and annealing at 70 °C for 7 min.

### Device Fabrication

Sky‐blue PeLEDs were fabricated with an architecture of ITO/PEDOT:PSS/CsPb(Cl/Br)_3_ perovskite/TPBi/LiF/Al, where ITO was used as the anode, PEDOT:PSS as an HTL, perovskite layer as the emitter, TPBi as an ETL, and LiF/Al as a bilayer cathode, respectively. The patterned ITO‐coated glass substrates were cleaned sequentially by sonication with detergent water, acetone, and ethanol for 5 min each, which were followed by the UV‐ozone treatment for 20 min prior to the spin‐coating of PEDOT:PSS. The PEDOT:PSS aqueous solution was modified by doping the APDO powder with a content of 2 mg mL^−1^. After depositing the perovskite films, the samples were transferred from the N_2_‐filled glove box into an interconnected high‐vacuum deposition system (base pressure <2 × 10^−6^ Torr) for the successive thermal evaporation of TPBi (40 nm), LiF (1 nm), and Al (100 nm) through shadow masks. The deposition rate and layer thickness were monitored with a quartz crystal oscillator. According to the cross‐sectional SEM characterization, the thicknesses of the PEDOT:PSS, perovskite, TPBi, and Al layers were measured at 28, 32, 42, and 98 nm on average, respectively. The effective areas of large‐area devices were 100 and 400 mm^2^ as determined by the overlap between ITO and Al electrodes. After completing the device fabrication, all the devices were transferred to the glove box for the encapsulation with a glass cap.

### Films and Device Characterization

The absorption spectra of perovskite films were recorded with an UV/vis/near‐IR spectrometer (Perkin Elmer Lambda 950). The absolute PLQYs of sky‐blue perovskite films modified by APDO with various concentrations were obtained through a C9920‐02G type fluorescence spectrophotometer (HAMAMASTU, Japan) with an integrating sphere excited at 365 nm at room temperature under nitrogen atmosphere. The morphologies of perovskite films and devices were characterized via scanning electron microscopy (SEM, Zeiss Supra 55). Contact angles were measured with a contact angle tester (DataPhysics instruments GmbH). The synchrotron‐based GIWAXS measurements were performed at the BL02U2 beamline of the Shanghai Synchrotron Radiation Facility using X‐ray with a wavelength of 1.24 Å and a grazing incidence angle of 0.2°, which were conducted by Pilatus3s 2M at an exposure time of 100 s at a distance of about 286 mm from the sample. 2D X‐ray diffraction (XRD) patterns were obtained using FIT2D software and displayed in the scattering vector q coordinates. The *J*–*V*–*L* characteristics and EL spectra of PeLEDs were measured simultaneously with a computer‐controlled programmable power source (Keithley model 2400) and a luminance meter/spectrometer (PhotoResearch PR670) in air ambience at room temperature. The angular light distribution was measured by placing samples on an autorotating stage. The EQEs of the devices were calculated by assuming a Lambertian emission profile, which were verified by the independent measurements of luminous flux with a photonic multichannel analyzer PMA‐12 (Hamamatsu C10027‐01) and an integrating sphere (Hamamatsu A10094). The impedance spectra were measured under a small AC signal with an amplitude of 300 mV at various DC voltages (Agilent 4294A).

## Conflict of Interest

The authors declare no conflict of interest.

## Supporting information

Supporting Information

Supplemental Video 1

Supplemental Video 2

## Data Availability

Research data are not shared.

## References

[1] S. D. Stranks , H. J. Snaith , Nat. Nanotechnol. 2015, 10, 391. 25947963 10.1038/nnano.2015.90

[2] M. Worku , A. Ben‐Akacha , T. B. Shonde , H. Liu , B. Ma , Small Sci. 2021, 1, 2000072. 10.1002/smsc.202000072 PMC11935801 40213161

[3] N. Wang , L. Cheng , R. Ge , S. Zhang , Y. Miao , W. Zou , C. Yi , Y. Sun , Y. Cao , R. Yang , Y. Wei , Q. Guo , Y. Ke , M. Yu , Y. Jin , Y. Liu , Q. Ding , D. Di , L. Yang , G. Xing , H. Tian , C. Jin , F. Gao , R. H. Friend , J. Wang , W. Huang , Nat. Photonics 2016, 10, 699.

[4] M. Yuan , L. N. Quan , R. Comin , G. Walters , R. Sabatini , O. Voznyy , S. Hoogland , Y. Zhao , E. M. Beauregard , P. Kanjanaboos , Z. Lu , D. H. Kim , E. H. Sargent , Nat. Nanotechnol. 2016, 11, 872. 27347835 10.1038/nnano.2016.110

[5] Z.‐K. Tan , R. S. Moghaddam , M. L. Lai , P. Docampo , R. Higler , F. Deschler , M. Price , A. Sadhanala , L. M. Pazos , D. Credgington , F. Hanusch , T. Bein , H. J. Snaith , R. H. Friend , Nat. Nanotechnol. 2014, 9, 687. 25086602 10.1038/nnano.2014.149

[6] K. Lin , J. Xing , L. N. Quan , F. Pelayo García de Arquer , X. Gong , J. Lu , L. Xie , W. Zhao , D. Zhang , C. Yan , W. Li , X. Liu , Y. Lu , J. Kirman , E. H. Sargent , Q. Xiong , Z. Wei , Nature 2018, 562, 245. 30305741 10.1038/s41586-018-0575-3

[7] Y.‐H. Kim , S. Kim , A. Kakekhani , J. Park , J. Park , Y.‐H. Lee , H. Xu , S. Nagane , R. B. Wexler , D.‐H. Kim , S. H. Jo , L. Martínez‐Sarti , P. Tan , A. Sadhanala , G.‐S. Park , Y.‐W. Kim , B. Hu , H. J. Bolink , S. Yoo , R. H. Friend , A. M. Rappe , T.‐W. Lee , Nat. Photonics 2021, 15, 148.

[8] Y. Cao , N. Wang , H. Tian , J. Guo , Y. Wei , H. Chen , Y. Miao , W. Zou , K. Pan , Y. He , H. Cao , Y. Ke , M. Xu , Y. Wang , M. Yang , K. Du , Z. Fu , D. Kong , D. Dai , Y. Jin , G. Li , H. Li , Q. Peng , J. Wang , W. Huang , Nature 2018, 562, 249. 30305742 10.1038/s41586-018-0576-2

[9] Y. Shen , C. Yan , K. Lin , Y. Zhao , S. Xu , B. Zhou , Z. Wei , K. Yan , Small Sci. 2021, 1, 2000077. 10.1002/smsc.202000077 PMC11935923 40213162

[10] X. Y. Qian , Y. Y. Tang , W. Zhou , Y. Shen , M. L. Guo , Y. Q. Li , J. X. Tang , Small Sci. 2021, 1, 2000048. 10.1002/smsc.202000048 PMC11935876 40213163

[11] Y. Jiang , C. Qin , M. Cui , T. He , K. Liu , Y. Huang , M. Luo , L. Zhang , H. Xu , S. Li , J. Wei , Z. Liu , H. Wang , G.‐H. Kim , M. Yuan , J. Chen , Nat. Commun. 2019, 10, 1868. 31015430 10.1038/s41467-019-09794-7 PMC6478869

[12] Z. Chu , Y. Zhao , F. Ma , C.‐X. Zhang , H. Deng , F. Gao , Q. Ye , J. Meng , Z. Yin , X. Zhang , J. You , Nat. Commun. 2020, 11, 4165. 32820166 10.1038/s41467-020-17943-6 PMC7441179

[13] P. Pang , G. Jin , C. Liang , B. Wang , W. Xiang , D. Zhang , J. Xu , W. Hong , Z. Xiao , L. Wang , G. Xing , J. Chen , D. Ma , ACS Nano 2020, 14, 11420. 32812732 10.1021/acsnano.0c03765

[14] F. Ye , W. Tang , F. Xie , M. Yin , J. He , Y. Wang , H. Chen , Y. Qiang , X. Yang , L. Han , Adv. Mater. 2017, 29, 1701440. 10.1002/adma.201701440 28707309

[15] D. P. Nenon , K. Pressler , J. Kang , B. A. Koscher , J. H. Olshansky , W. T. Osowiecki , M. A. Koc , L.‐W. Wang , A. P. Alivisatos , J. Am. Chem. Soc. 2018, 140, 17760. 30501174 10.1021/jacs.8b11035

[16] H. Cho , S.‐H. Jeong , M.‐H. Park , Y.‐H. Kim , C. Wolf , C.‐L. Lee , J. H. Heo , A. Sadhanala , N. Myoung , S. Yoo , S. H. Im , R. H. Friend , T.‐W. Lee , Science 2015, 350, 1222. 26785482 10.1126/science.aad1818

[17] L. P. Cheng , J. S. Huang , Y. Shen , G. P. Li , X. K. Liu , W. Li , Y. H. Wang , Y.‐Q. Li , Y. Jiang , F. Gao , C. S. Lee , J. X. Tang , Adv. Opt. Mater. 2019, 7, 1801534.

[18] C. Bi , Z. Yao , X. Sun , X. Wei , J. Wang , J. Tian , Adv. Mater. 2021, 2006722. 10.1002/adma.202006722 33629762

[19] Z. Li , Z. Chen , Y. Yang , Q. Xue , H.‐L. Yip , Y. Cao , Nat. Commun. 2019, 10, 1027. 30833581 10.1038/s41467-019-09011-5 PMC6399279

[20] F. Yuan , C. Ran , L. Zhang , H. Dong , B. Jiao , X. Hou , J. Li , Z. Wu , ACS Energy Lett. 2020, 5, 1062.

[21] Q. Wang , X. Wang , Z. Yang , N. Zhou , Y. Deng , J. Zhao , X. Xiao , P. Rudd , A. Moran , Y. Yan , J. Huang , Nat. Commun. 2019, 10, 5633. 31822670 10.1038/s41467-019-13580-w PMC6904584

[22] H. Wang , X. Gong , D. Zhao , Y.‐B. Zhao , S. Wang , J. Zhang , L. Kong , B. Wei , R. Quintero‐Bermudez , O. Voznyy , Y. Shang , Z. Ning , Y. Yan , E. H. Sargent , X. Yang , Joule 2020, 4, 1977.

[23] Y. Shen , K.‐C. Shen , Y.‐Q. Li , M. Guo , J. Wang , Y. Ye , F.‐M. Xie , H. Ren , X. Gao , F. Song , J.‐X. Tang , Adv. Funct. Mater. 2021, 31, 2006736.

[24] L. Zhang , X. Yang , Q. Jiang , P. Wang , Z. Yin , X. Zhang , H. Tan , Y. Yang , M. Wei , B. R. Sutherland , E. H. Sargent , J. You , Nat. Commun. 2017, 8, 15640. 28589960 10.1038/ncomms15640 PMC5467226

[25] X. Meng , J. Lin , X. Liu , X. He , Y. Wang , T. Noda , T. Wu , X. Yang , L. Han , Adv. Mater. 2019, 31, 1903721. 10.1002/adma.201903721 31495977

[26] Y. Shen , M.‐N. Li , Y. Li , F.‐M. Xie , H.‐Y. Wu , G.‐H. Zhang , L. Chen , S.‐T. Lee , J.‐X. Tang , ACS Nano 2020, 14, 6107. 32223190 10.1021/acsnano.0c01908

[27] F. Wang , W. Geng , Y. Zhou , H.‐H. Fang , C.‐J. Tong , M. A. Loi , L.‐M. Liu , N. Zhao , Adv. Mater. 2016, 28, 9986. 27677653 10.1002/adma.201603062

[28] Y. Liu , L. Cai , Y. Xu , J. Li , Y. Qin , T. Song , L. Wang , Y. Li , L. K. Ono , Y. Qi , B. Sun , Nano Energy 2020, 78, 105134.

[29] K. Zhu , S. Cong , Z. Lu , Y. Lou , L. He , J. Li , J. Ding , N. Yuang , M. H. Rümmeli , G. Zou , J. Power Sources 2019, 428, 82.

[30] M. Ban , Y. Zou , J. P. H. Rivett , Y. Yang , T. H. Thomas , Y. Tan , T. Song , X. Gao , D. Credgington , F. Deschler , H. Sirringhaus , B. Sun , Nat. Commun. 2018, 9, 3892. 30250032 10.1038/s41467-018-06425-5 PMC6155305

[31] A. Dey , J. Ye , A. De , E. Debroye , S. K. Ha , E. Bladt , A. S. Kshirsagar , Z. Wang , J. Yin , Y. Wang , L. N. Quan , F. Yan , M. Gao , X. Li , J. Shamsi , T. Debnath , M. Cao , M. A. Scheel , S. Kumar , J. A. Steele , M. Gerhard , L. Chouhan , K. Xu , X. Wu , Y. Li , Y. Zhang , A. Dutta , C. Han , I. Vincon , A. L. Rogach , A. Nag , A. Samanta , B. A. Korgel , C.‐J. Shih , D. R. Gamelin , D. H. Son , H. Zeng , H. Zhong , H. Sun , H. V. Demir , I. G. Scheblykin , I. Mora‐Seró , J. K. Stolarczyk , J. Z. Zhang , J. Feldmann , J. Hofkens , J. M. Luther , J. Pérez‐Prieto , L. Li , L. Manna , M. I. Bodnarchuk , M. V. Kovalenko , M. B. J. Roeffaers , N. Pradhan , O. F. Mohammed , O. M. Bakr , P. Yang , P. Müller‐Buschbaum , P. V. Kamat , Q. Bao , Q. Zhang , R. Krahne , R. E. Galian , S. D. Stranks , S. Bals , V. Biju , W. A. Tisdale , Y. Yan , R. L. Z. Hoye , L. Polavarapu , ACS Nano 2021, 10.1021/acsnano.0c08903.

[32] J. Ye , M. M. Byranvand , C. O. Martínez , R. L. Z. Hoye , M. Saliba , L. Polavarapu , Angew. Chem., Int. Ed. 2021, 60, 2. 10.1002/anie.202102360 PMC8518834 33730428

[33] M. Xu , Q. Peng , W. Zou , L. Gu , L. Xu , L. Cheng , Y. He , M. Yang , N. Wang , W. Huang , J. Wang , Appl. Phys. Lett. 2019, 115, 041102.

[34] H. Zhang , F. Ye , W. Li , R. S. Gurney , D. Liu , C. Wang , T. Xiong , ACS Appl. Energy Mater. 2019, 2, 3336.

[35] I. Zarazúa , S. Sidhik , T. Lopéz‐Luke , D. Esparza , E. De la Rosa , J. Reyes‐Gomez , I. Mora‐Seró , G. Garcia‐Belmonte , J. Phys. Chem. Lett. 2017, 8, 6073. 29186659 10.1021/acs.jpclett.7b02848

[36] D. Yao , X. Mao , X. Wang , Y. Yang , N. D. Pham , A. Du , P. Chen , L. Wang , G. J. Wilson , H. Wang , ACS Appl. Mater. Interfaces 2020, 12, 6651. 31918551 10.1021/acsami.9b19908

[37] J. Takahashia , J. Appl. Phys. 2019, 125, 245501.

[38] L. Yang , X. Li , Q. Yang , S. Wang , H. Tian , J. Ding , L. Wang , Adv. Funct. Mater. 2021, 31, 2007686.

[39] F. Yuan , Y.‐K. Wang , G. Sharma , Y. Dong , X. Zheng , P. Li , A. Johnston , G. Bappi , J. Z. Fan , H. Kung , B. Chen , M. I. Saidaminov , K. Singh , O. Voznyy , O. M. Bakr , Z.‐H. Lu , E. H. Sargent , Nat. Photonics 2020, 14, 171.

[40] L. Zuo , H. Guo , D. W. deQuilettes , S. Jariwala , N. De Marco , S. Dong , R. DeBlock , D. S. Ginger , B. Dunn , M. Wang , Y. Yang , Sci. Adv. 2017, 3, e1700106. 28845446 10.1126/sciadv.1700106 PMC5567759

[41] M. K. Gangishetty , S. Hou , Q. Quan , D. N. Congreve , Adv. Mater. 2018, 30, 1706226. 10.1002/adma.201706226 29575250

[42] A. Fakharuddin , W. Qiu , G. Croes , A. Devižis , R. Gegevičius , A. Vakhnin , C. Rolin , J. Genoe , R. Gehlhaar , A. Kadashchuk , V. Gulbinas , P. Heremans , Adv. Funct. Mater. 2019, 29, 1904101.

[43] Y.‐H. Kim , H. Cho , J. H. Heo , T.‐S. Kim , N. Myoung , C.‐L. Lee , S. H. Im , T.‐W. Lee , Adv. Mater. 2015, 27, 1248. 25420784 10.1002/adma.201403751

[44] J.‐S. Kim , R. H. Friend , I. Grizzi , J. H. Burroughes , Appl. Phys. Lett. 2005, 87, 023506.

