# Supplementary material for: Interfacial “Anchoring Effect” Enables Efficient Large‐Area Sky‐Blue Perovskite Light‐Emitting Diodes
Source: Adv Sci (Weinh). 2021 Aug 28;8(19):2102213. doi: 10.1002/advs.202102213 (PMC8498857; doi:10.1002/advs.202102213)
Supplement: Supplementary file 1 — Supporting Information [file ADVS-8-2102213-s002.pdf]

© 2021 Wiley-VCH GmbH

ADVANCED SCIENCE
Open Access

Supporting Information

for *Adv. Sci.*, DOI: 10.1002/advs.advs202102213

Interfacial "Anchoring Effect" Enables Efficient Large-Area Sky-Blue Perovskite Light-Emitting Diodes

Yang Shen, Jing-Kun Wang, Yan-Qing Li,* Kong-Chao Shen, Zhen-Huang Su, Li Chen, Ming-Lei Guo, Xiao-Yi Cai, Feng-Ming Xie, Xiao-Yan Qian, Xingyu Gao, Ivan S. Zhidkov, and Jian-Xin Tang*

Supporting Information

Interfacial "Anchoring Effect" Enables Efficient Large-Area Sky-Blue Perovskite Light-Emitting Diodes

Yang Shen, Jing-Kun Wang, Yan-Qing Li,* Kong-Chao Shen, Zhen-Huang Su, Li Chen, Ming-Lei Guo, Xiao-Yi Cai, Feng-Ming Xie, Xiao-Yan Qian, Xingyu Gao, Ivan S. Zhidkov, and Jian-Xin Tang*

Y. Shen, J. K. Wang, Dr. K. C. Shen, L. Chen, M. L. Guo, X. Y. Cai, F. M. Xie, X. Y. Qian, Prof. J. X. Tang
Jiangsu Key Laboratory for Carbon-Based Functional Materials & Devices
Institute of Functional Nano & Soft Materials (FUNSOM)
Soochow University
Suzhou 215123, China
E-mail: jxtang@suda.edu.cn (J.X. Tang)

Prof. Y. Q. Li
School of Physics and Electronic Science
Ministry of Education Nanophotonics & Advanced Instrument Engineering Research Center
East China Normal University
Shanghai, 200062, China
E-mail: yqli@phy.ecnu.edu.cn (Y.Q. Li)

Z. H. Su, Prof. X. Gao
Key Laboratory of Interfacial Physics and Technology
Shanghai Institute of Applied Physics
Shanghai Synchrotron Radiation Facility, Zhangjiang Laboratory
Chinese Academy of Sciences
Shanghai, 201204, China

Dr. I. S. Zhidkov
Institute of Physics and Technology
Ural Federal University
Mira 19 str., 620002, Yekaterinburg, Russia

Prof. J. X. Tang
Macao Institute of Materials Science and Engineering (MIMSE)
Macau University of Science and Technology
Taipa 999078, Macau SAR, China

Supplementary Measurements

X-ray and ultraviolet photoelectron spectroscopies (XPS and UPS) measurements were performed with an ultrahigh vacuum photoemission spectroscopy system (Kratos AXIS Ultra^{DLD}). XPS spectra were collected by using a monochromatic Al Kα source (1486.6 eV) with a total instrumental energy resolution of 500 meV. UPS spectra were obtained with a He I ($h\nu$ = 21.22 eV) gas discharge lamp for excitation and a total instrumental energy resolution of 100 meV. Steady-state photoluminescence (PL) spectra were obtained with a FluoroMax-4 fluorescence spectrometer (Horiba Jobin Yvon) under the ambient environment. Transient PL decay measurements were performed with a Quantaurus-Tau fluorescence lifetime spectrometer (C11367-32, Hamamatsu Photonics) in ambient with a 373 nm pulsed laser (pulse width of 100 ps and repetition rate of 5 KHz). Transient electroluminescence (EL) decay curves of PeLEDs were measured under an electrical excitation with a pulse width of 20 μs (duty cycle of 10%) generated by a pulse generator (keysight 81150A). Nuclear magnetic resonance (NMR) measurements were carried out with a Bruker AVANCE III type NMR spectrometer by using deuterated N, N-dimethylsulfoxide-d_6 (DMSO-d_6) as the solvent. The Fourier transform infrared spectroscopy (FTIR) measurements were conducted by using absorption infrared spectrometer (VERTX 70).

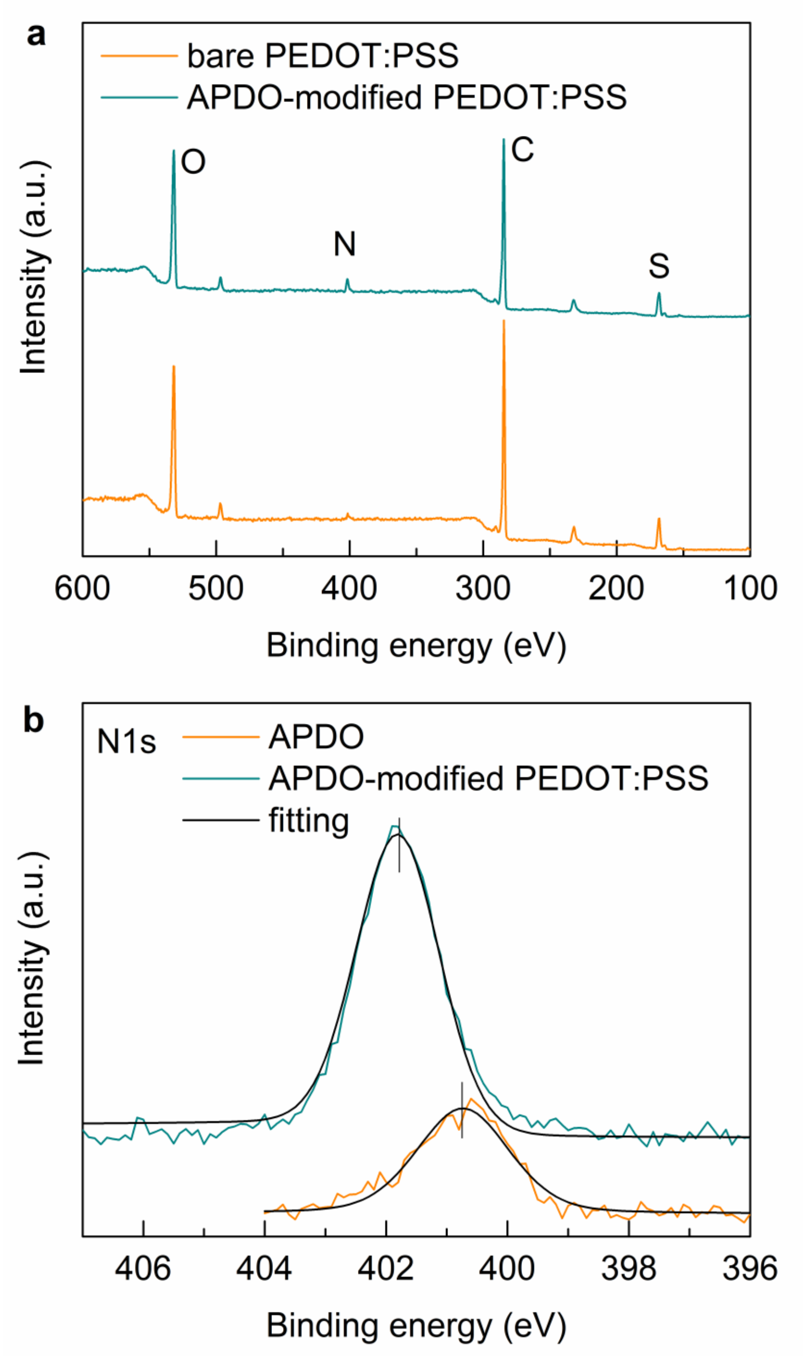

Figure S1. Modification of the PEDOT:PSS HTL with APDO. a) XPS survey scans of pristine and APDO-modified PEDOT:PSS films. b) XPS spectra of N *1s* core level of APDO and APDO-modified PEDOT:PSS.

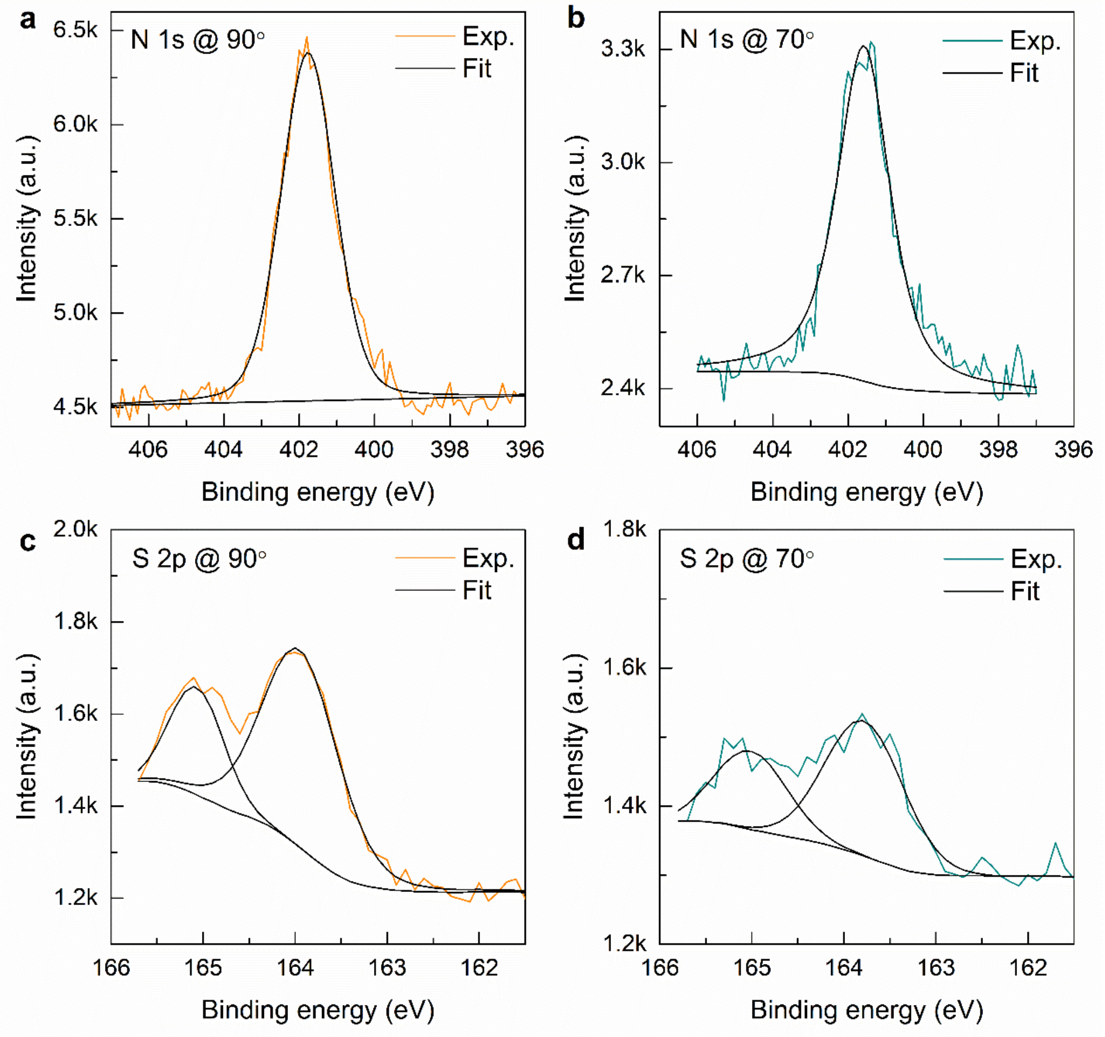

Figure S2. Distribution characterization of APDO in the annealed PEDOT:PSS HTLs. XPS spectra of N *1s* core level detected at the photoelectron emission angles of (a) 90° and (b) 70° relative the sample surface. XPS spectra of S *2p* core level detected at the photoelectron emission angles of (c) 90° and (d) 70° relative the sample surface.

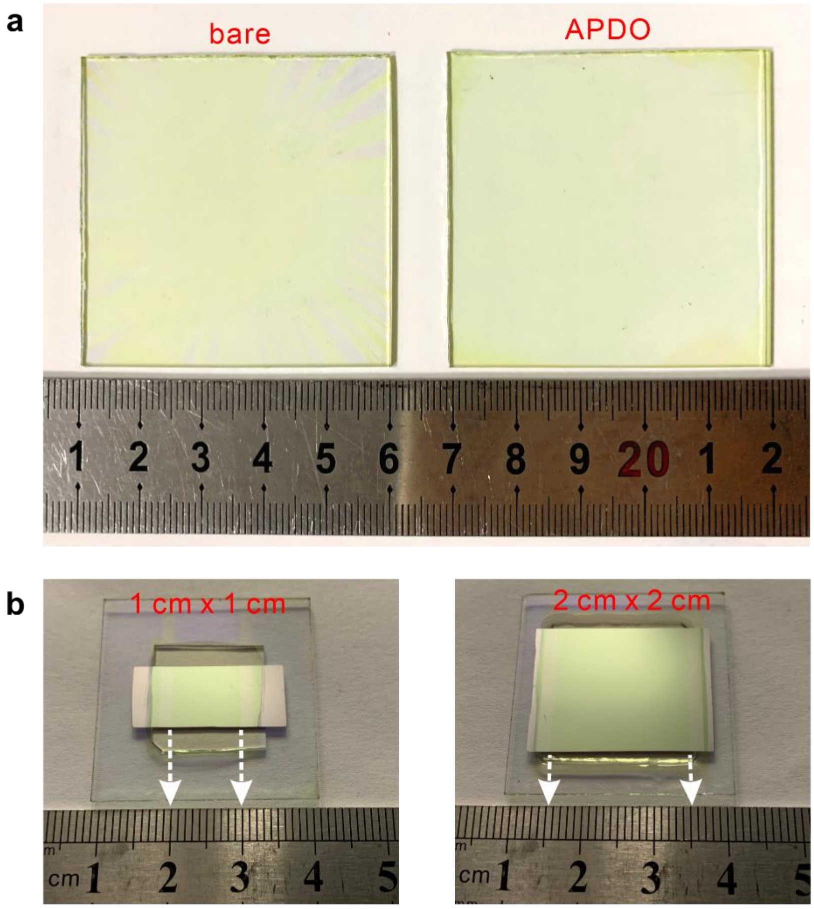

Figure S3. Photographs of large-area perovskite films and PeLEDs. a) Perovskite films deposited on bare and APDO-modified PEDOT:PSS HTLs. b) The APDO-modified PeLEDs with an emitting area of 10 mm × 10 mm and 20 mm × 20 mm, respectively.

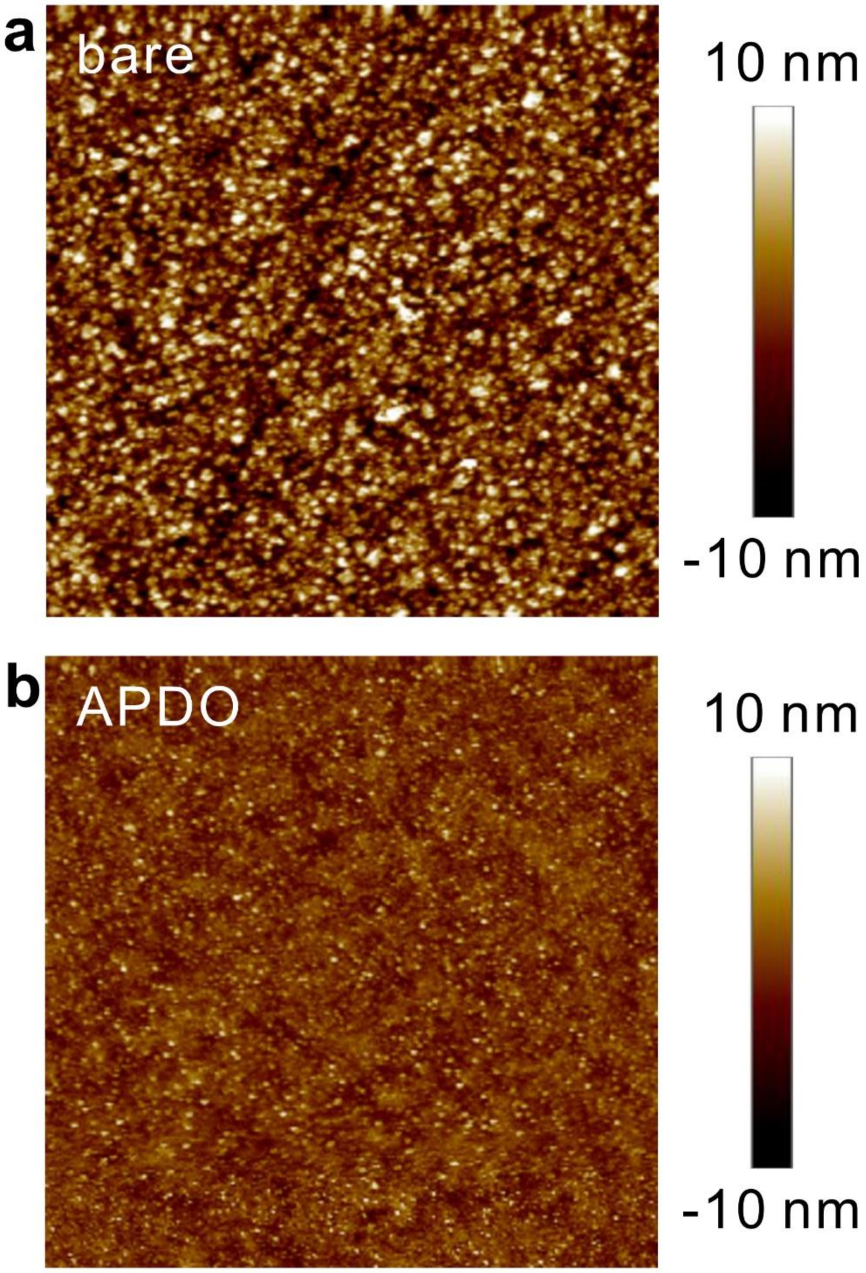

Figure S4. Surface roughness characterization of perovskite films. AFM images in height mode of perovskite films deposited on a) bare and b) APDO-modified PEDOT:PSS HTLs

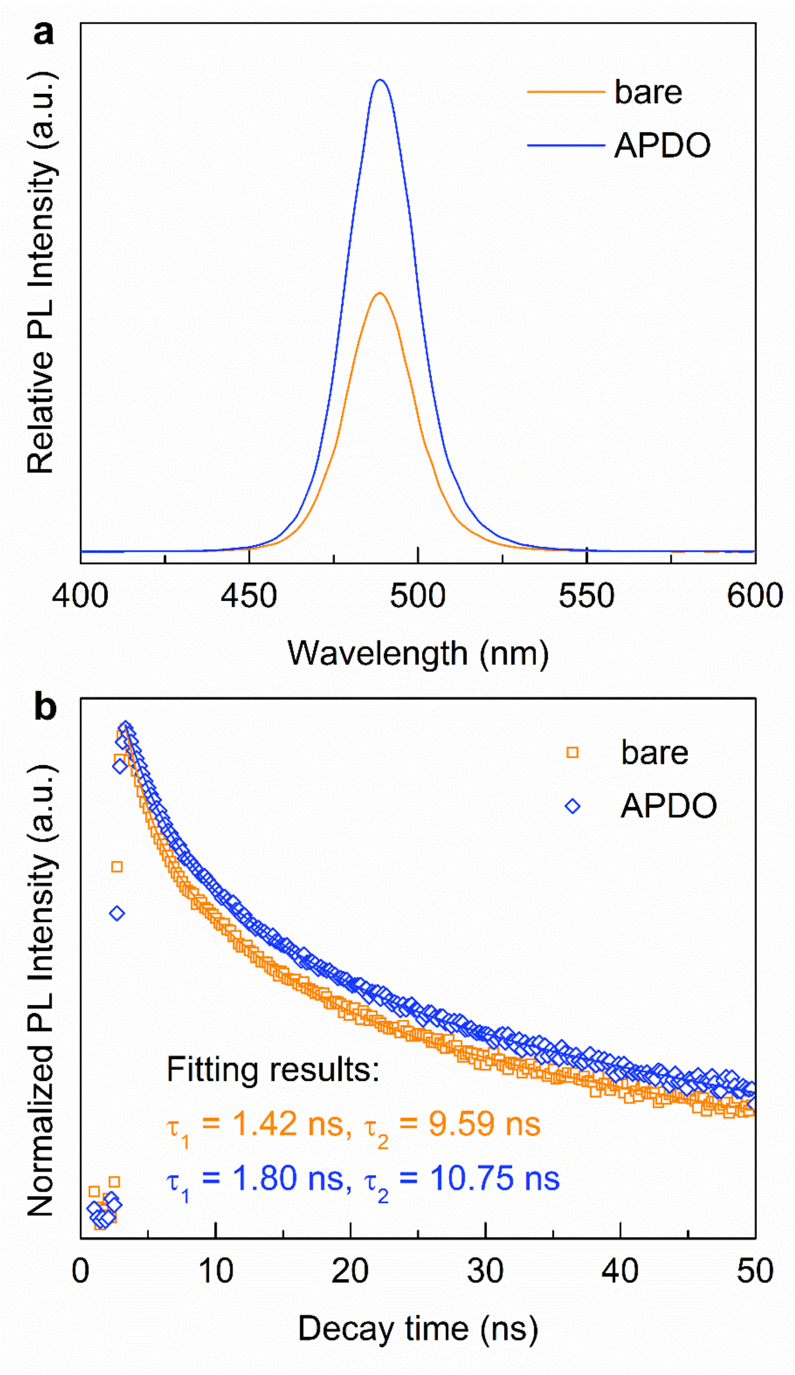

Figure S5. Optical characterizations of perovskite films. a) Relative steady PL intensity and b) transient PL decay curves of perovskite films deposited on bare and APDO-modified PEDOT:PSS HTLs.

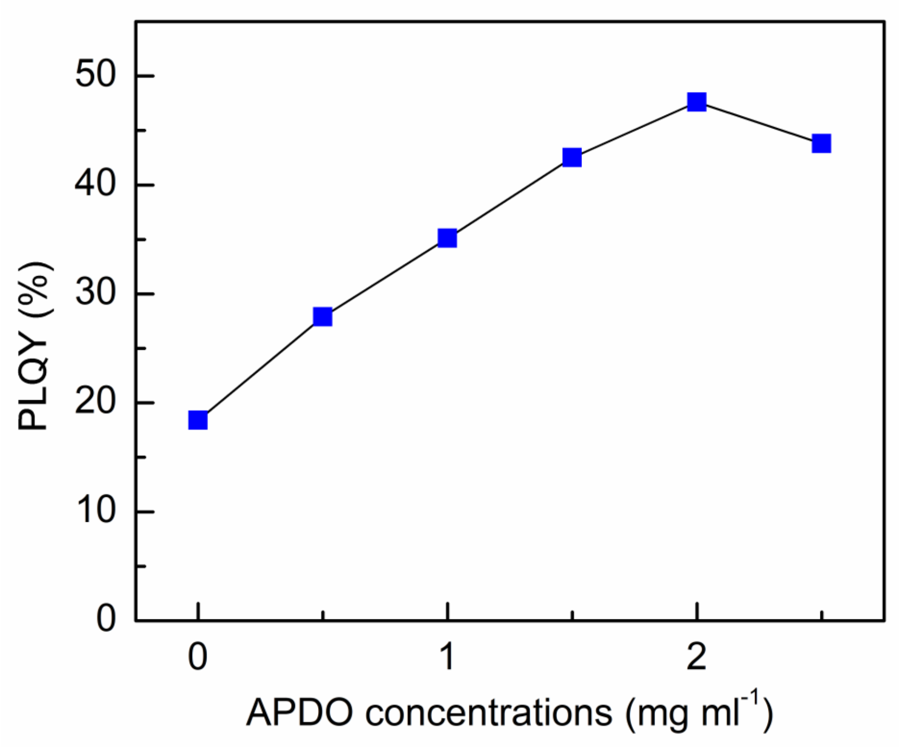

Figure S6. Photoluminescence quantum yields (PLQYs) of the sky-blue perovskite films deposited on the PEDOT:PSS layers with the APDO-doping concentrations of 0, 0.5, 1.0, 1.5, 2.0, and 2.5 mg ml^{-1}, respectively. The PLQY data is collected in Table S2.

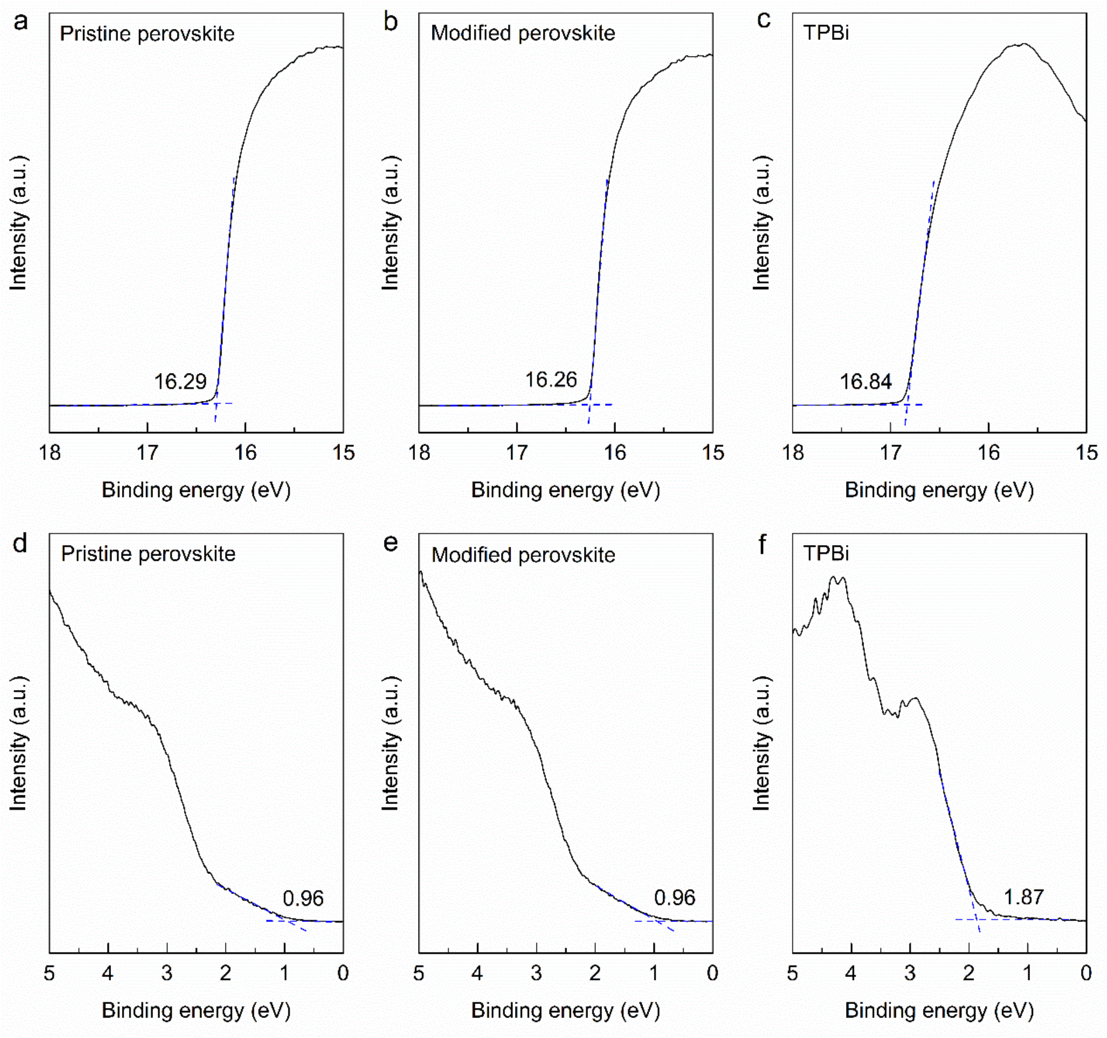

Figure S7. Energy-level characterization. (a-c) Secondary-electron cutoff and (d-f) onset regions of the UPS spectra of (a,d) pristine perovskite films, (b,e) APDO-modified perovskite films and (c,f) TPBi fims.

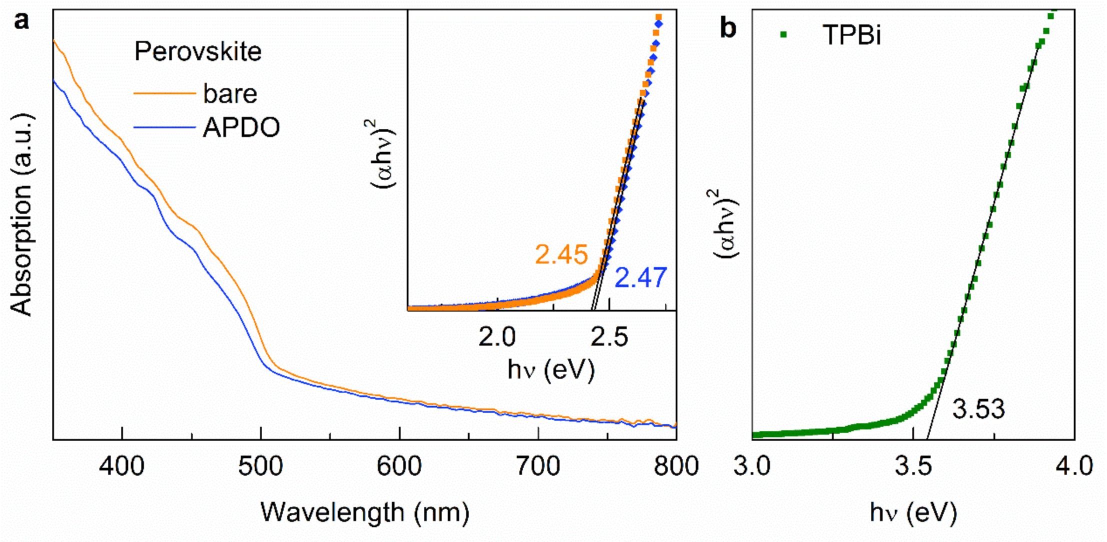

Figure S8. Optical bandgap characterization. a) Absorption spectra of perovskite films deposited on bare and APDO-modified PEDOT:PSS HTLs. The inset shows the Tauc analyses. b) Tauc analysis of the TPBi film.

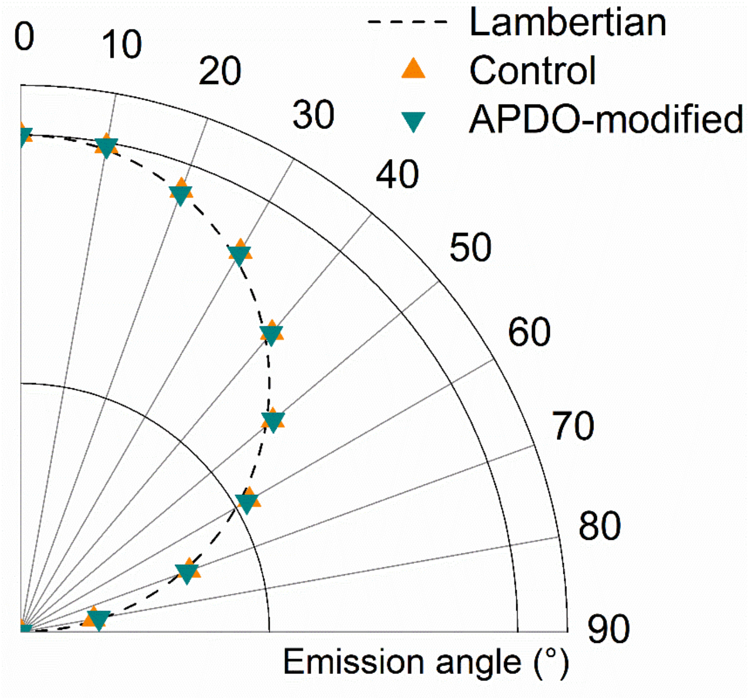

Figure S9. Angle dependence of the EL intensity profiles of control and APDO-modified PeLEDs.

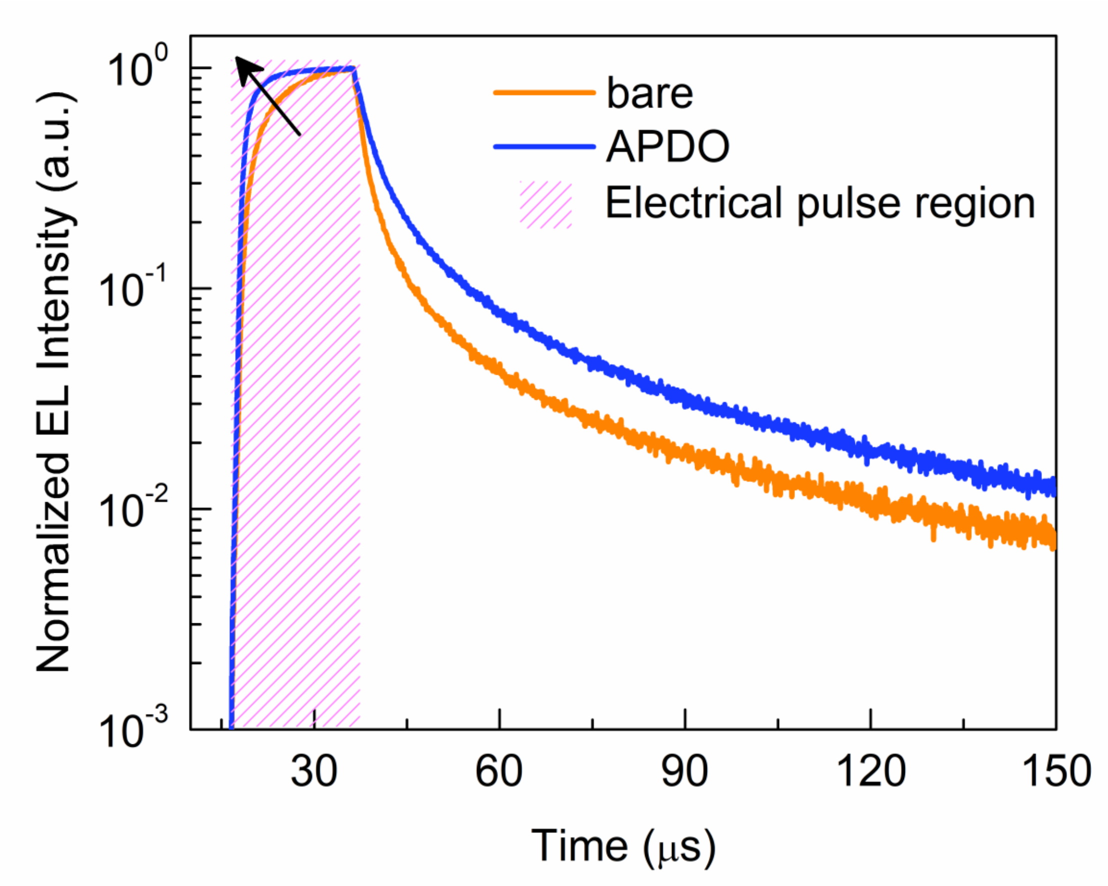

Figure S10. Transient EL decay curves with 10% duty cycle for bare and APDO-modified PeLEDs.

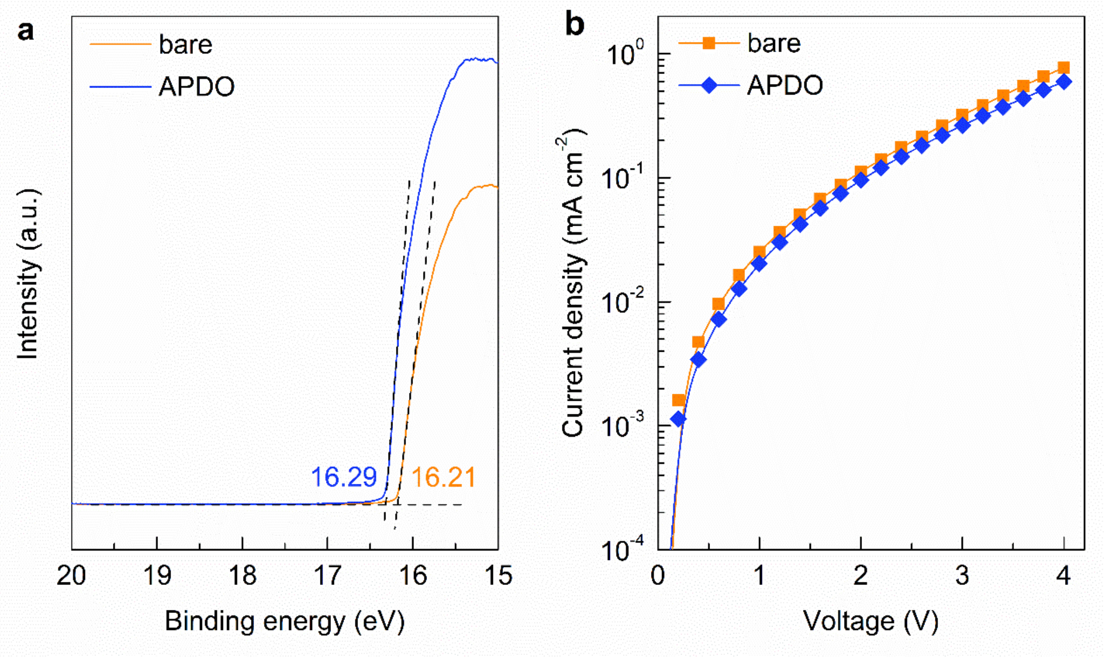

Figure 11. Hole-transport characterizations of bare and APDO-modified PEDOT:PSS HTLs. a) Secondary-electron cutoff region of the UPS spectra of the PEDOT:PSS HTLs. b) J-V curves of perovskite-free hole-only devices with a structure of ITO/PEDOT:PSS/CBP/MoOx/Al.

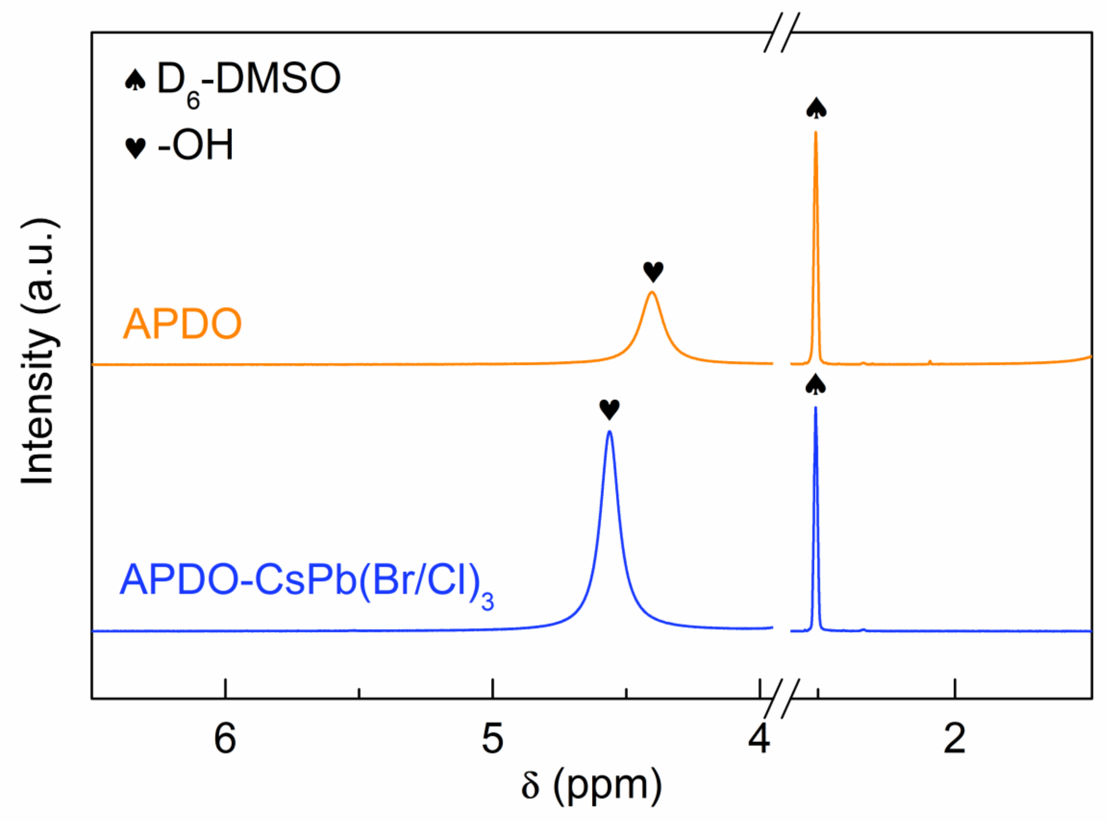

Figure S12. ^{1}H nuclear magnetic resonance (NMR) spectra of APDO and APDO-modified $CsPb(Br/Cl)_3$ with deuterated N, N-dimethylsulfoxide-d_6 (DMSO-d_6) as the solvent. The proton signal arising from −OH group in pure APDO shifts from δ = 4.40 ppm to δ = 4.56 ppm with the addition of $CsPb(Br/Cl)_3$ perovskite, which is indicative of the O−H···Br/Cl^- hydrogen bonding interaction.

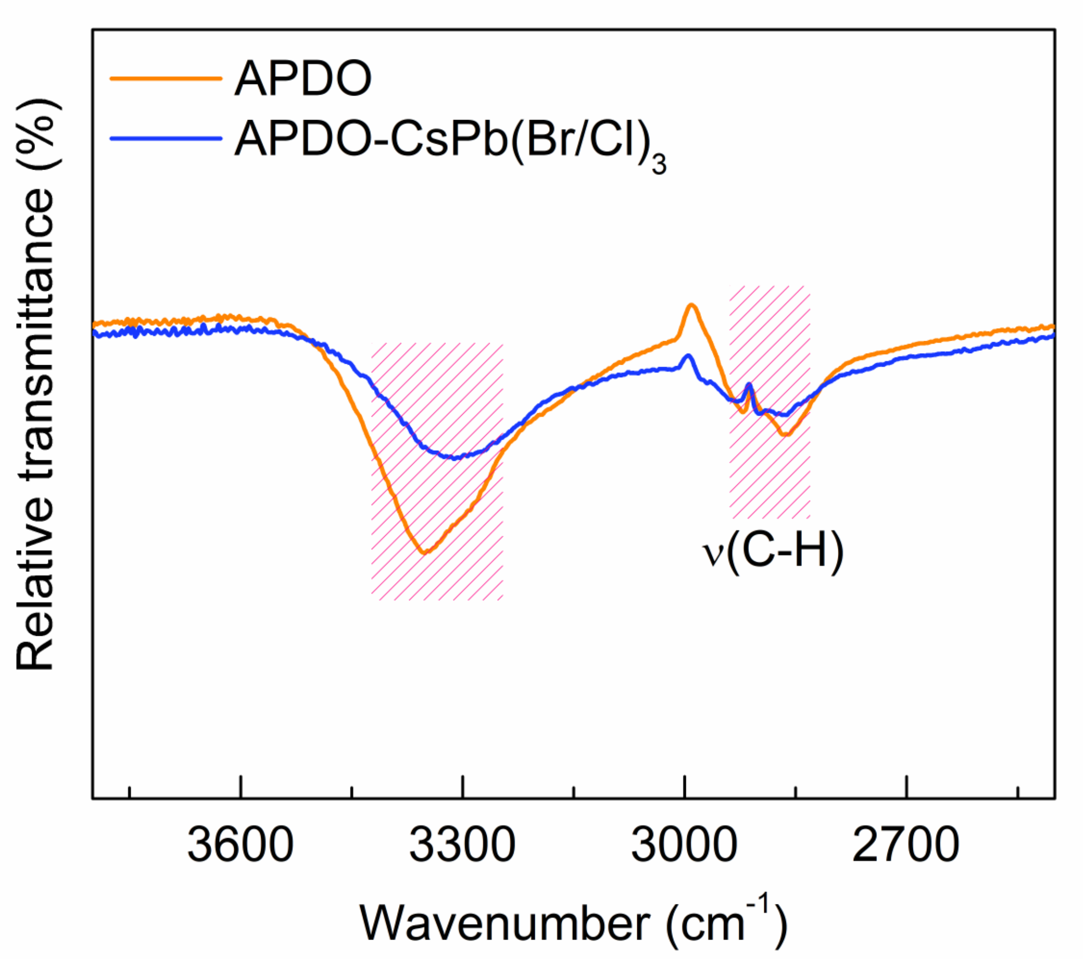

Figure S13. Fourier transform infrared spectroscopy (FTIR) relative transmittance spectra of APDO molecule and APDO-modified $CsPb(Br/Cl)_3$. The band position shifts from 3350 cm^{-1} to 3310 cm^{-1} by mixing $CsPb(Br/Cl)_3$ perovskite with APDO, which is on account of the formation of O−H···Br/Cl hydrogen bond with the halide sites in the PbX_2 octahedrons (X = Br/Cl).

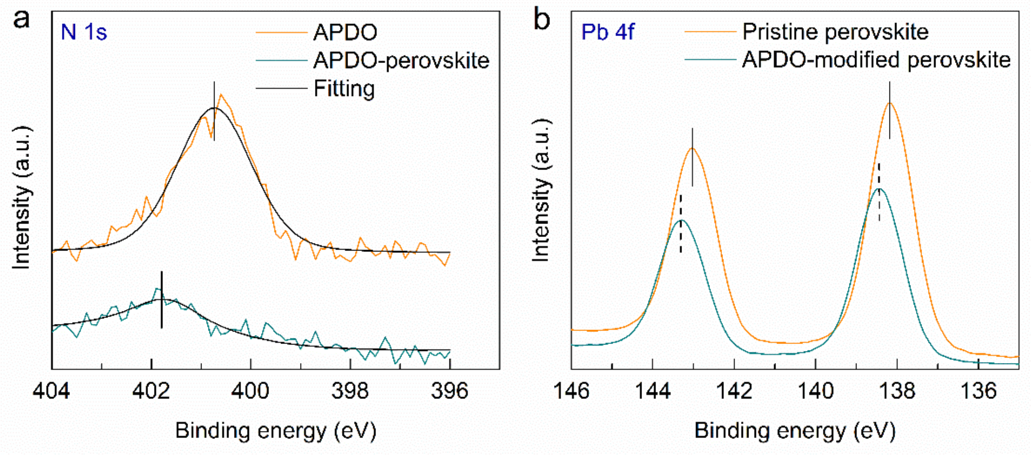

Figure 14. XPS spectra for APDO, pristine and APDO-modified perovskite films. a) XPS spectra for N *1s* core level. b) XPS spectra for Pb *4f* core level.

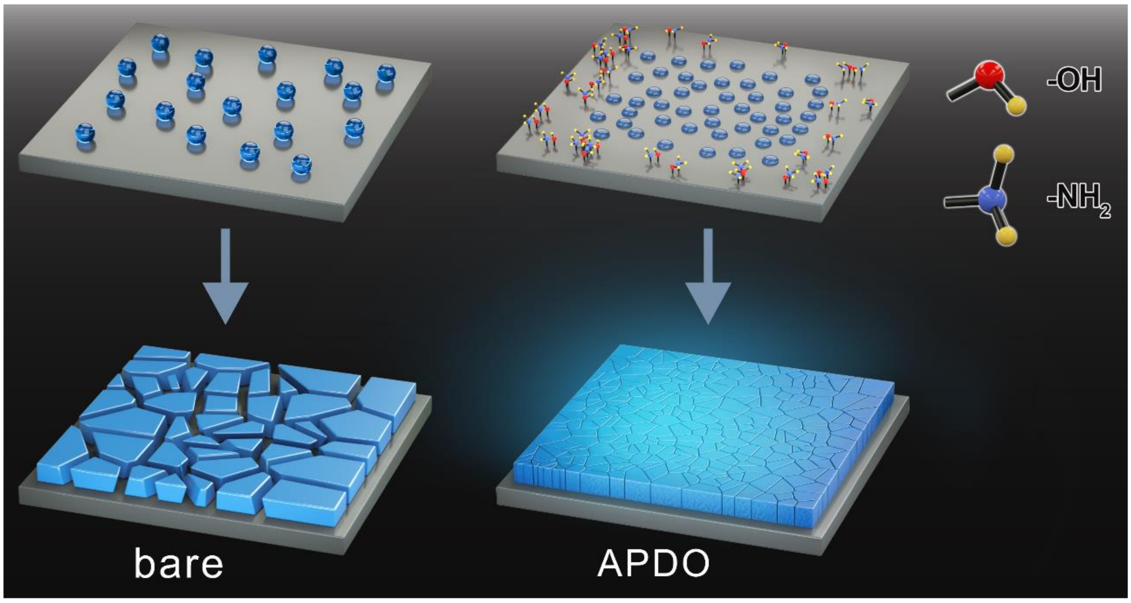

Figure S15. Schematic diagram of the sky-blue perovskite crystallization evolution on bare and APDO-modified substrates.

Table S1. Peak areas of N *1s* and S *2p* core levels in the APDO-modified PEDOT:PSS HTL and their elemental ratios at different photoemission angles.

Peak area				N/S Ratio (R)*	
90°		70°		90°	70°
N *1s*	S *2p*	N *1s*	S *2p*	N/S	N/S
3640.1	657.62	2031.5	320.51	7.72	8.85

* N/S ratio R was obtained with the equation of R = $(A_N/S_N)/(A_S/S_S)$, where A_N and A_S are the fitted peak area of N *1s* and S *2p*, and S_N (=0.477) and S_S (=0.666) are the corresponding atomic sensitivity factors for the measurements using Al Kα X-ray light, respectively.

Table S2. PLQYs of sky-blue perovskite films and external quantum efficiencies (EQEs) of their correlated PeLEDs with various APDO-doping concentrations. The emitting area of PeLEDs is 100 mm^2.

C ($mg\ ml^{-1}$)	0	0.5	1.0	1.5	2.0	2.5
PLQYs (%)	18.4	27.9	35.1	42.5	47.6	43.8
EQEs (%)	3.4	5.1	6.6	7.8	9.2	8.1

Note: C refers to the APDO-doping concentrations.

Table S3. Fitted R_{rec} data in the IS measurement for bare and APDO-modified PeLEDs.

R_{rec} (Ω cm^2)	1.0 V	2.0 V	3.0 V	3.5 V	4.0 V	5.0 V
Bare	2.4×10^5	2.3×10^5	1.8×10^5	1.2×10^5	2.5×10^4	880
APDO	3.8×10^5	3.6×10^5	1.9×10^5	4.6×10^4	5.5×10^3	289

Table S4. Summary of key EL parameters for sky-blue PeLEDs.

Device structures	EL [nm]	Max L [cd m^{-2}]	EQE [%]	Refs
ITO/PEDOT:PSS/PVK/ BA:CsPb(Br/Cl)$_3$/TPBi/Al	487	3340	6.2	1
ITO/NiO_x/TFB/PVK/ $Cs_xFA_{1–x}PbBr_3$/TPBi/LiF/Al	483	~700	9.5	2
ITO/PEDOT:PSS/ PEA:CsPb($Br_{0.5}Cl_{0.5}$)$_3$/TPBi/LiF/Al	485	9040	11.0	3
ITO/PEDOT:PSS/ $PEA_2(Cs_{1-x}EA_xPbBr_3)_2PbBr_4$/TPBi/LiF/Al	488	2191	12.1	4
ITO/PVK/$PEA_xPA_{2–x}(CsPbBr_3)_{n–1}PbBr_4$/ TPBi/LiF/Al	486	513	10.1	5
ITO/NiO_x/PTAA/PVK/ PEA:CsPb($Br_xCl_{1–x}$)$_3$/TPBi/LiF/Al	488	~2000	11.7	6
ITO/LiF/(Cs/Rb/FA/PEA/K)Pb(Cl/Br)$_3$/ LiF/Bphen/LiF/Al	484	4015	2.01	7
ITO/PEDOT:PSS/CsPb(Br_xCl_{1x})$_3$/ TPBi/LiF/Al	486	1390	12.8	8
ITO/NiO_x/PVK/PEA:CsPb($Br_xCl_{1–x}$)$_3$/ TPBi/LiF/Al	477	2180	11.0	9
ITO/PEDOT:PSS/CsPb(Br_xCl_{1x})$_3$/ TPBi/LiF/Al	490	1775	9.2	This work

Reference

[1] P. Vashishtha, M. Ng, S. B. Shivarudraiah, J. E. Halpert, *Chem. Mater*. **2019**, *31*, 83.

[2] Y. Liu, J. Cui, K. Du, H. Tian, Z. He, Q. Zhou, Z. Yang, Y. Deng, D. Chen, X. Zuo, Y. Ren, L. Wang, H. Zhu, B. Zhao, D. Di, J. Wang, R. H. Friend, Y. Jin, *Nat. Photonics* **2019**, *13*, 760.

[3] Q. Wang, X. Wang, Z. Yang, N. Zhou, Y. Deng, J. Zhao, X. Xiao, P. Rudd, A. Moran, Y. Yan, J. Huang, *Nat. Commun*. **2019**, *10*, 5633.

[4] Z. Chu, Y. Zhao, F. Ma, C.-X. Zhang, H. Deng, F. Gao, Q. Ye, J. Meng, Z. Yin, X. Zhang, J. You, *Nat Commun*. **2020**, *11*, 4165.

[5] Z. Ren, J. Yu, Z. Qin, J. Wang, J. Sun, C. C. S. Chan, S. Ding, K. Wang, R. Chen, K. S. Wong, X. Lu, W.-J. Yin, W. C. H. Choy, *Adv. Mater*. **2021**, *33*, 2005570.

[6] P. Pang, G. Jin, C. Liang, B. Wang, W. Xiang, D. Zhang, J. Xu, W. Hong, Z. Xiao, L. Wang, G. Xing, J. Chen, D. Ma, *ACS Nano* **2020**, *14*, 11420.

[7] F. Yuan, C. Ran, L. Zhang, H. Dong, B. Jiao, X. Hou, J. Li, Z. Wu, *ACS Energy Lett*. **2020**, *5*, 1062.

[8] Y. Shen, H.-Y. Wu, Y.-Q. Li, K.-C. Shen, X. Gao, F. Song, J.-X. Tang, *Adv. Funct. Mater.* **2021**, 2103870.

[9] M. Karlsson, Z. Yi, S. Reichert, X. Luo, W. Lin, Z. Zhang, C. Bao, R. Zhang, S. Bai, G. Zheng, P. Teng, L. Duan, Y. Lu, K. Zheng, T. Pullerits, C. Deibel, W. Xu, R. H. Friend, F. Gao, *Nat. Commun*. **2021**, *12*, 361.
